# Functional and Morphological Changes in the Visual Pathway in Patients with Graves’ Orbitopathy

**DOI:** 10.3390/jcm11144095

**Published:** 2022-07-15

**Authors:** Agnieszka Jagiełło-Korzeniowska, Agata Bałdys-Waligórska, Alicja Hubalewska-Dydejczyk, Bożena Romanowska-Dixon

**Affiliations:** 1 Department of Ophthalmology and Ocular Oncology, Jagiellonian University Medical College, 31-501 Kraków, Poland; bozena.romanowska-dixon@uj.edu.pl; 2 Department of Endocrinology and Internal Medicine, Faculty of Health Science and Medicine, Andrzej Frycz Modrzewski Krakow University, 30-705 Kraków, Poland; awalig@cm-uj.krakow.pl; 3 Department of Endocrinology, Jagiellonian University Medical College, 30-688 Kraków, Poland; alicja.hubalewska-dydejczyk@uj.edu.pl

**Keywords:** Graves’ Orbitopathy, dysthyroid optic neuropathy, visual evoked potential, pattern electroretinogram, optical coherence tomography, retinal nerve fiber layer, retinal ganglion cell complex

## Abstract

Background: The aim of the study was to perform a functional and structural evaluation of the anterior visual pathway in patients with Graves’ Orbitopathy (GO) using electrophysiological tests and OCT, as well as to identify potential parameters that could be useful in detecting early optic nerve damage. Methods: 47 GO patients were enrolled in the study and divided into three groups, depending on their disease severity: Group 1 with mild GO, Group 2 with moderate-to-severe GO, and Group 3 with dysthyroid optic neuropathy (DON). Pattern visual evoked potential (PVEP), flash visual evoked potential (fVEP), pattern electroretinogram (pERG), and optical coherence tomography (OCT) findings were compared between the groups. Results: In the DON Group (Group 3), N75, P100, and P2 latencies were significantly extended, whereas P100, P50, and N95 amplitudes were significantly reduced as compared to the non-DON group (Groups 1 and 2). Group 3 also had significantly thinner peripapillary retinal nerve fiber layer (RNFL) and macular ganglion cell complex (GCC). In Group 2, as compared to Group 1, P100 amplitudes were significantly reduced for all check sizes, while P100 latency was elongated for the check size of 0.9°. Group 2 also had a significantly thinner average GCC and GCC in the superior quadrant. Conclusions: Electrophysiological examinations may be of use in diagnosis of DON. OCT findings and electrophysiological responses vary in patients with different GO severity. Including regular electrophysiological evaluation and OCT in the examination of patients with GO could be of benefit. However, more research is needed to establish the true significance of pVEP, fVEP, pERG, and OCT in monitoring patients with GO.

## 1. Introduction

Graves’ Orbitopathy (GO) is the most common extrathyroidal expression of Graves’ disease (GD). It is an autoimmune, antibody-mediated disorder leading to the inflammation and remodeling of orbital tissues. Excessive production of glycosaminoglycans (GAGs), adipogenesis, oedema, and inflammatory infiltration induce muscle enlargement and orbital fat expansion within the constrained space of the orbit. This process may result in apical crowding and in the direct compression of the optic nerve or its blood supply [1].

Dysthyroid optic neuropathy (DON) is a rare condition with an insidious onset, affecting approximately 5–8% of patients with Graves’ Orbitopathy. It requires urgent management as it can potentially lead to irreversible visual loss [2,3]. A thorough ophthalmological examination, including visual acuity and color vision assessment, pupillary test, perimetry, and fundus examination is needed to make a diagnosis of dysthyroid optic neuropathy. A relative pupillary defect (RAPD) and optic nerve edema are highly specific of DON. However, RAPD may be absent in 50% of DON cases due to bilateral optic nerve involvement. Optic nerve oedema, in turn, is not present in 45–80% of DON cases [4,5]. Vision loss, which is the most common sign of DON, is highly unspecific. It may just as well result from ocular surface abnormalities associated with GO [6]. However, even a mild decrease in visual acuity or blurred vision should not be underestimated, as 50–70% of patients with confirmed DON have visual acuity of 20/40 or better [3]. The diagnosis of DON may be delayed at the subclinical stage, which is why electrophysiological assessment of the optic nerve and retinal ganglion cell function comprising flash and pattern visual evoked potentials (fVEP, pVEP) along with pattern electroretinogram (pERG) may be of use in monitoring patients with Graves’ Orbitopathy. While pattern visual evoked potentials have been extensively studied, there are very few studies analyzing fVEP in patients with DON [7]. Several researchers have reported that patients with DON have prolonged N75 and P100 latencies as well as a decreased P100 amplitude [8,9]. Tsaloumas et al. observed a smaller amplitude of P2 in DON patients [9]. It has been postulated that electrophysiological examinations, in particular pVEP and pERG, can be used to detect the presence of subclinical DON. There are several studies comparing pVEP and pERG in GO patients without confirmed DON with healthy controls, but the results are inconsistent [10,11,12,13]. In addition to the above-mentioned electrophysiological techniques for evaluating the optic nerve and retinal ganglion cell function, morphological assessment by means of optical coherence tomography (OCT) may also be of use in monitoring patients with GO. OCT enables an analysis of the optic nerve head (ONH), retinal ganglion cell complex (GCC), and peripapillary retinal nerve fiber layer (RNFL). However, studies analyzing OCT findings in GO patients are scarce and their results are contradictory [14,15,16]. To the best of our knowledge, no attempts have been made so far to perform a simultaneous functional and structural assessment of the anterior visual pathway in patients with Graves’ Orbitopathy.

The objective of our research was to perform a functional and morphological examination of the visual pathway in patients with Graves’ Orbitopathy using pVEP, fVEP, pERG, and OCT, as well as to compare the results in patients with different degrees of severity of the disease.

## 2. Materials and Methods

47 patients with Graves’ Orbitopathy were enrolled in the study, including 13 men (28%) and 34 women (72%). The patients’ mean age was 52.1 years ± 14.8 SD. The research protocol was approved by the Jagiellonian University Bioethical Committee (Approval No. 1072.6120.163.2017) and the study adhered to the tenets of the Declaration of Helsinki. All patients signed a written informed consent. The exclusion criteria were other ocular diseases, high myopia greater than—6.0 D, intraocular pressure above 21 mmHg, neurological diseases (such as multiple sclerosis, Alzheimer’s disease, Parkinson’ s disease, and brain tumors), diabetes with polyneuropathy or diabetic retinopathy, and other diseases that could have an impact on electrophysiological or OCT examinations. The ophthalmological examination included visual acuity assessment, color vision assessment using Ishihara tables, tonometry, biomicroscopic anterior segment examination, cover test and Hess’s screen, indirect fundus examination, Hertel exophthalmometry, and manual kinetic Goldmann perimetry. GO severity was evaluated according to the European Group on Graves’ Orbitopathy (EUGOGO) classification (Table 1) [17]. Patients suspected of DON had orbital imagining with the measurements of particular extraocular muscles performed. Six patients had magnetic resonance imagining (MRI) and thirteen patients had Computed Tomography (CT) of the orbits. The electrophysiological examinations were performed using an EP-1000 device by TOMEY GmbH (Nuremberg, Germany). All examinations were conducted without pupil dilation. For the pVEP, the full-field, pattern-reversal protocol was used with four different check sizes, i.e., 0.4°, 0.9°, 1.5°, and 2.5°. Given that most patients with Graves’ Orbitopathy have severe ocular surface abnormalities that influence visual acuity and cause blurred vision, we decided against using a small check size of 0.25°. We applied a mid check size of 0.4° instead and a large check size of 0.9° as stated in the International Society for Clinical Electrophysiology of Vision (ISCEV) recommendations [18]. Additionally, very large checks of 1.5° and 2.5° were used in the study. The electrodes were placed as follows: the active electrode at Oz- 5 cm above the inion, the reference electrode at Fz- 11 cm above the nasion, and the ground electrode at the earlobe. Monocular stimulation was performed with full optical correction. Flash visual evoked potentials were elicited using the Ganzfeld bowl. The placement of electrodes was the same as in the pVEP examination. Monocular stimulation was performed with the fellow eye patched by a black obturator. We performed a transient pattern electroretinogram using the Ganzfeld bowl for stimulation. Binocular recording was conducted with an appropriate optical correction for the test distance. Fiber recording electrodes were positioned at the lower conjunctival fornix after topical anesthesia with proxymetacaine. The reference electrodes were placed on the skin at the outer canthus of each ipsilateral eye and the ground electrode was attached at the earlobe. The conditions of all the electrophysiological examinations were in line with ISCEV recommendations [18,19].

Optical coherence tomography was performed after pupil dilation. An RTVue OCT device (Model RT-100, version 6.3, OPTOVUE, Fremont, CA, USA) was used. An RNFL scan of 3.45 diameters, centered on the optic nerve head, was performed. The analysis of the peripapillary retinal nerve fiber layer thickness was presented in µm as Average RNFL, Superior RNFL, and Inferior RNFL.

The greatest number of ganglion cells is found in the central 8 degrees of the retina; therefore, a macular ganglion cell complex (GCC) analysis was performed. The GCC consists of 3 layers of the retina, i.e., the retinal nerve fiber layer, the retinal ganglion cell layer, and the inner plexiform layer. The GCC protocol comprised 15 vertical scans and 1 horizontal scan covering an area of 7 by 7 mm, localized 1mm temporally from the macula. The analysis of GCC thickness was presented in µm as Average GCC, Superior GCC, and Inferior GCC. Additionally, the Focal Loss Volume (FLV) and Global Loss Volume (GLV) were analyzed.

The patients were divided according to the EUGOGO severity classification: into mild (Group 1), moderate-to-severe (Group 2), and sight-threatening (Group 3). The groups comprised 16, 23, and 8 patients, respectively. We compared the results of electrophysiological examinations and OCT of patients with no clinical evidence of DON (Groups 1 and 2) with patients with confirmed DON (Group 3). Subsequently, we compared patients with mild GO (Group 1) with patients with moderate-to-severe GO (Group 2) in terms of their electrophysiological responses and OCT parameters.

### Statistical Analysis

A Student’s t-test for independent samples was used to test differences between groups of patients. To test if the variances of two populations are equal, an F-test for equality of two variances was used additionally. Age and sex differences of patients between the groups were estimated using Student’s *t*-test and Mann–Whitney’s u-test (for independent samples).

## 3. Results

### 3.1. Comparison between GO Patients with No Clinical Evidence of DON (Groups 1 and 2) and Patients with Confirmed DON (Group 3)

The non-DON Group (Groups 1 and 2) comprised of 39 patients (9 men and 30 women). The mean age of patients in this group was 50.4 ± 14.7. Group 3 comprised 8 patients (4 men and 4 women). The mean age of patients in this group was 60.5 ± 12.8. The differences between the groups in terms of patients’ sex and age were statistically insignificant (*p* = 0.2402 and *p* = 0.0771, respectively)

#### 3.1.1. Pattern Visual Evoked Potentials

We compared the latencies of N75 and P100, as well as the amplitudes of P100, in confirmed DON patients (Group 3) with GO patients with no clinical evidence of DON (Groups 1 and 2). The comparison revealed a significant increase in N75 and P100 latencies for all check sizes and a significant reduction of P100 amplitudes for all check sizes except for check size of 1.5° in Group 3 (Table 2). The differences in latencies were much more pronounced than the differences in P100 amplitudes.

#### 3.1.2. Flash Visual Evoked Potentials

As for fVEP, the mean latency of P2 was 133.4 ± 16 ms vs. 118.0 ± 13.7 ms (*p* = 0.0001) in the DON group (Group 3) and in the non-DON Group (Groups 1 and 2), respectively. There was no statistically significant difference in the P2 amplitude between the two groups (10.1 ± 7.0 vs. 12.0 ± 6.5 *p* = 0.2828).

#### 3.1.3. Pattern Electroretinogram

There was a significant reduction in N95 and P50 amplitudes in Group 3 (Table 3). P50 latency was significantly delayed whereas the latency of N95 was shorter in Group 3 (Table 3).

#### 3.1.4. Optical Coherence Tomography

A macular ganglion cell complex analysis revealed significant differences between the groups. The average GCC as well as inferior and superior quadrant GCC were significantly thinner in Group 3 (Table 4). In contrast, FLV and GLV indices representing the focal and global ganglion cell loss volume, respectively, were significantly greater in Group 3. The average peripapillary RNFL thickness as well as the RNFL thickness in the superior and inferior quadrants were also significantly smaller in Group 3; however, these differences were not so pronounced (Table 4).

### 3.2. Comparison between MILD GO Patients (Group 1) and MODERATE-TO-SEVERE GO Patients (Group 2)

Group 1 comprised of 16 patients (1 man and 15 women). The mean age of patients in this group was 47.1 ± 16.2.

Group 2 comprised of 23 patients (8 men and 15 women). The mean age of patients in this group was 52.7 ± 13.5. There were more men in Group 2 (*p* = 0.0420), which was to be expected since men tend to have more severe GO. The percentage of men in the groups in this study increased along with GO severity. The age differences between the groups were statistically insignificant (*p* = 0.2490).

#### 3.2.1. Pattern Visual Evoked Potentials

We found that P100 amplitudes were significantly reduced in patients with moderate-to-severe GO for all check sizes. There was a statistically elongated P100 latency for check size of 0.9° in those patients. However, the differences in P100 amplitudes were much more pronounced. (Table 5). There were no differences in N75 latencies between the groups.

#### 3.2.2. Flash Visual Evoked Potentials

The groups did not differ in terms of P2 amplitude and latency.

#### 3.2.3. Pattern Electroretinogram

The groups did not differ in terms of P50 or N95 amplitudes and latencies.

#### 3.2.4. Optical Coherence Tomography

The groups differed in terms of the average GCC and GCC in the superior quadrant.

There were no statistically significant differences in terms of inferior quadrant GCC, peripapillary RNFL, FLV, or GLV (Table 6).

## 4. Discussion

In the present study, we performed a thorough investigation of the functional and structural changes of the visual pathway in patients with Graves’ Orbitopathy. We found significant differences in electrophysiological responses and OCT parameters in patients with different GO severity.

A visual evoked potential is produced by activated neurons of the occipital cortex. It is generated in response to visual stimulation: a flashing light (fVEP) or an alternating checkboard pattern (pVEP). The fVEP consists of several positive and negative components, among which P2 is the most commonly used in clinical assessment. We found a significantly prolonged P2 latency in patients with dysthyroid optic neuropathy, as compared to GO patients without evident signs of DON (133.4 ± 16 ms vs. 118.0 ± 13.7 ms), but we did not find any differences in P2 amplitudes between the groups (10.1 ± 7.0 vs. 12.0 ± 6.5 *p* = 0.2828). Our results are in contradiction to those of Tsaloumas et al., who reported no difference in P2 latency between the DON and thyroid-associated orbitopathy (TAO) groups (112.0 ± 4.46 ms vs. 110.1 ± 2.65 ms) but found a significantly smaller P2 amplitude in DON patients (6.83 ± 0.92 ms vs. 12.4 ± 1.05 µV). Tsaloumas et al. admitted, however, that in their study, reductions in amplitude occurred more frequently than delays as compared to other studies [9]. Our results are in line with Setälä et al., who identified a significant increase in the latency of the main positive fVEP component in patients requiring orbital decompression [20]. Studies concerning the fVEP in patients with GO are scarce, however, and they are also difficult to compare due to the heterogenous conditions under which the examinations were conducted. The fVEP is less sensitive and more variable between individuals than the pVEP, which might be the reason why it is less commonly used in research. However, it has some advantages over the pVEP, being less dependent on the macular input and patients’ cooperation. It may be particularly useful in some patients with GO, who have severe ocular surface changes, poor visual acuity, or symptoms like tearing and diplopia, which impede proper concentration and make it difficult to obtain a reliable response to checkboard stimuli [21,22].

We did not find any differences in fVEP responses between patients with mild and moderate-to-severe GO.

The pVEP is a triphasic waveform with a negative N75, positive P100 and negative N135 components. P100 latency and amplitude are the most commonly used parameters in clinical practice. In the present study, pVEP examination in patients with DON revealed significantly extended N75 and P100 latencies, as well as significantly reduced P100 amplitudes, as compared to GO patients without DON symptoms. This was in accordance with previous studies [7,9,23]. While comparing the mild GO group and the moderate-to-severe GO group, we found notably reduced P100 amplitudes and prolonged P100 latency for 0.9 check size in the latter group. We did not find any changes in N75 latencies. Previous reports in this matter are inconsistent. Shawkat et al. and Tsaloumas et al. found no differences in pVEP responses between GO patients and normal controls, whereas Salvi et al. and Acaroğlu et al. found significant delays of the P100 component [7,9,10,24]. Pawłowski et al. described both N75 and P100 latency increase in GO patients without any symptoms of optic nerve dysfunction in comparison to healthy controls [12]. In contrast, Ambrosio et al. emphasized the importance of the P100 amplitude as a sensitive indicator of compressive nerve damage in patients with GO and concomitant glaucoma [8]. The discrepancies found in the earlier reports may result from the fact that the authors failed to take account of GO severity. There were some attempts to correlate proptosis with electrophysiological findings; however, proptosis only represents one of the factors assessed in GO severity classifications [12,25]. We believe that patients with a less severe disease may demonstrate no changes in electrophysiological responses, their optic nerve may not be endangered, and they may not differ from normal controls. The situation, however, may change insidiously along with GO progression.

It was shown in several studies that spatial frequency (check size) has an impact on pVEP responses [26]. Some authors emphasize that higher spatial frequencies target more foveal retinal ganglion cells, whereas larger checks stimulate better peripheral vision [21,27,28]. We used additional low spatial frequencies in our protocol to see if they could be of use in monitoring patients with GO. We were unable to confirm that using larger check sizes when performing a pVEP examination in patients with Graves’ Orbitopathy is more beneficial in everyday practice than the standard procedure. Also, pVEP latencies for all check sizes were prolonged in patients with DON in comparison to patients without clinical features of DON, while pVEP amplitudes were reduced in the moderate-to-severe GO group as compared to mild GO group regardless of the check size used for the examination. The P100 latency in patients with moderate-to-severe GO was prolonged only for a check size of 0.9° as compared to patients with mild GO. Therefore, higher spatial frequencies seem to be more suitable for detecting subclinical, functional changes in the optic nerve in patients with GO, which is in line with other studies [8,29]. However, we do not find that longer pVEP protocols with different check sizes are of benefit in patients with GO, who often experience severe ocular surface abnormalities.

The pattern visual evoked potential is not specific to optic nerve damage as it depends on good macular function. Pattern electroretinogram is a complementary technique to visual evoked potentials. It enables simultaneous assessment of retinal ganglion cell and macular function. The P50 component of the pERG is generated by inner- and outer-retinal neurons, whereas the N95 component represents retinal ganglion cell function [29,30]. To the best of our knowledge there are very few studies analyzing pERG in patients with GO and no studies whatsoever describing pERG in patients with DON.

We found significantly reduced P50 and N95 amplitudes in patients with DON as compared to GO patients without confirmed DON. The P50 latency was significantly prolonged, whereas the N95 latency was reduced in those patients. The differences in latencies, however, were much less pronounced than those in amplitudes (Table 2) and were absent when the right and the left eye were compared separately. Pawłowski et al. postulated that the P50 component could be used as a marker of early optic nerve dysfunction in GO patients, as in their study, its amplitude was reduced in GO patients without evident DON [13]. We did not find any differences in PERG parameters while comparing patients with moderate-to-severe GO (Group 2) and patients with mild GO (Group 1). Our results are in line with those obtained by Spadea et al. They reported that patients with very severe GO had a significant reduction in pERG and pVEP amplitudes and a significant increase in pVEP latencies, whereas patients with less severe GO only demonstrated a reduction in the P100 amplitude [11]. They suggested that a pVEP amplitude reduction represented a decrease in normally functioning optic nerve fibers, thus being more sensitive in early optic nerve dysfunction. Meanwhile, they attributed the pVEP delay and pERG amplitude reduction to retrograde axonal degeneration. Some authors emphasize that dysthyroid optic neuropathy is not only a compressive optic neuropathy characterized by direct nerve compression impairing axoplasmic flow, but also a sort of ischemic optic neuropathy with compromised vascular perfusion due to enlarged ocular muscles and increased intraorbital pressure [13]. The pVEP amplitude reduction in patients with moderate-to-severe GO may be due to early ischemic changes in the optic nerve [29,30,31,32,33,34]. The P50 component of the pERG may also occasionally be affected in eyes with ischemic optic neuropathy, suggesting a dysfunction at the level of macular photoreceptors or bipolar cells [29].

In patients with DON, a significant reduction in P50 amplitude may be secondary to retrograde damage of the retinal ganglion cells or may be due to ischemic changes in the outer retinal layers. The elongated P50 latency, which is characteristic of macular involvement, is more suggestive of the latter [35,36]. We cannot explain the N95 latency reduction in patients with DON.

Our analysis of OCT parameters in GO patients revealed that patients with DON had a significantly thinner average peripapillary RNFL, as well as RNFL in the upper and lower quadrants. Our results are in agreement with the study by Park et al., who reported a significantly smaller mean temporal peripapillary RNFL thickness in patients with long-lasting DON (≥6 months) in comparison to healthy controls and patients with acute DON [14]. In contrast, Meirovitch et al. found increased peripapillary RNFL thickness in superior, inferior, and nasal quadrants in patients with GO [16]. Peripapillary RNFL is not a reliable indicator of axon loss in patients with Graves’ Orbitopathy. The compression of the optic nerve by overgrown orbital tissues may impede axoplasmic flow, leading to the swelling of the axons. RNFL thickening may be misleading in accurate neuronal loss assessment [37]. The discrepancies between previous studies may result from the fact that patients at different stages of the disease were enrolled in the studies. Increased RNFL thickness in patients with GO may indicate disc edema, which may not be detectable in fundus examination, whereas RNFL thinning in patients with DON may indicate the onset of the optic nerve atrophy.

When comparing the macular ganglion cell complex in DON patients and in GO patients without any clinical signs of DON, we found significant differences in all the parameters. The average GCC, as well as inferior and superior quadrant GCC, were significantly thinner in DON patients. Both the foveal loss volume (FLV) and the global loss volume (GLV) were significantly increased in DON patients. Our results are in line with the study by Romano et al., who compared patients with optic nerve compression due to GO with normal controls and found a significantly thinner average GCC, inferior GCC, as well as significantly thinner RNFL in the superior and inferior quadrant in the first group [38]. Since the retinal ganglion cell complex is not affected by axon swelling, its analysis is better suited for assessing axon loss in the course of GO than RNFL [37]. In our study, the differences in GCC between patients with DON and GO patients were much more pronounced than those in RNFL thickness. The differences in RNFL were not statistically significant when analyzed for the left and for the right eye separately. Additionally, while comparing OCT parameters in patients with mild GO and moderate-to-severe GO, the only statistically significant differences that we found between the groups were in average GCC and GCC in the superior quadrant.

Orbital MRI was not performed in each patient, which is a limitation of this study as such scans would have revealed the factors which contributed to the changes found in the electrophysiological tests. Moreover, an additional comparison with a healthy control group would have given us a more complete picture of the morphological and functional changes in patients with GO.

## 5. Conclusions

Dysthyroid optic neuropathy may have an insidious onset. Hence, assessing optic nerve function in electrophysiological examinations may be of use in the diagnosis of DON. In our study, we observed significantly reduced P100, P50, and N95 amplitudes, as well as significant increased N75, P100, and P2 latencies in patients with DON. Combining pVEP with pERG may be helpful in distinguishing between changes resulting from macular and ganglion cell dysfunction. fVEP may be useful in patients with severe eye surface disorders and corneal or lens opacities. Previous studies have shown that changes in electrophysiological and OCT results are likely to be present in GO patients without clinical signs of DON. In this study, patients with moderate-to-severe GO had significantly reduced P100 amplitudes, a significantly longer P100 latency for the check size of 0.9°, and reduced average and superior GCC thickness as compared to patients with mild GO. Therefore, we conclude that electrophysiological responses and OCT parameters vary in patients with different GO severity. Including regular electrophysiological evaluation and OCT in the examination of patients with GO could be of benefit. However, more research is needed to establish the true significance of pVEP, fVEP, pERG, and OCT in monitoring patients with GO.

## Figures and Tables

**Table 1:** Graves’ Orbitopathy (GO) severity assessment according to European Group on Graves’ Orbitopathy (EUGOGO).

EUGOGO Classification:
**Mild GO:** Patients whose features of GO have only a minor impact on daily life that have insufficient impact to justify immunomodulation or surgical treatment. They usually have one or more of the following: minor lid retraction (<2 mm), mild soft-tissue involvement, exophthalmos <3 mm above normal for race and gender, no or intermittent diplopia, and corneal exposure responsive to lubricants
**Moderate-to-severe GO:** Patients without sight-threatening GO whose eye disease has sufficient impact on daily life to justify the risks of immunosuppression (if active) or surgical intervention (if inactive). They usually have two or more of the following: lid retraction ≥ 2 mm, moderate or severe soft-tissue involvement, exophthalmos ≥ 3 mm above normal for race and gender, inconstant or constant diplopia
**Sight-threatening (very severe) GO:** Patients with dysthyroid optic neuropathy and/or corneal breakdown

**Table 2:** A comparison of visual evoked potential (VEP) components between GO patients with no clinical evidence of dysthyroid optic neuropathy (DON) (Groups 1 and 2) and patients with confirmed DON (Group 3).

Parameter	Check Size	GROUPS 1 and 2 NON-DON (Mean ± SD)	GROUP 3 DON (Mean ± SD)	*p-Value*
N75 latency (ms)	*0.4°*	69.1 ± 6.6	79 ± 11.9	*p = 0.0001* *
	*0.9°*	64.3 ± 6.7	75.6 ± 8.2	*p < 0.0001* *
	*1.5°*	62.8 ± 9.0	79.6 ± 15.2	*p < 0.0001* *
	*2.5°*	64.1 ± 12.3	83.5 ± 16.6	*p < 0.0001* *
P100 latency (ms)	*0.4°*	95.8 ± 8.6	111.2 ± 13.3	*p < 0.0001* *
	*0.9°*	94.5 ± 8.5	110.3 ± 15.0	*p < 0.0001* *
	*1.5°*	94.0 ± 10.7	110.6 ± 13.8	*p < 0.0001* *
	*2.5°*	94.0 ± 12.5	110.8 ± 14.4	*p = 0.0001* *
P100 amplitude (µV)	*0.4°*	11.5 ± 6.0	7.5 ± 3.8	*p = 0.0317* *
	*0.9°*	10.7 ± 5.9	7.4 ± 3.1	*p = 0.0414* *
	*1.5°*	9.9 ± 5.0	7.2 ± 2.9	*p = 0.0607*
	*2.5°*	9.0 ± 4.0	5.7 ± 3.4	*p = 0.0089* *

* Statistically significant results are marked with an asterisk.

**Table 3:** A comparison of pattern electroretinogram (PERG) components between GO patients with no clinical evidence of DON (Groups 1 and 2) and patients with confirmed DON (Group 3).

Parameter	GROUPS 1 and 2 NON-DON (Mean ± SD)	GROUP 3 DON (Mean ± SD)	*p-Value*
N95 amplitude (µV)	8.3 ± 4.1	4.0 ± 2.3	*p = 0.0001* *
P50 amplitude (µV)	6.3 ± 2.8	3.5 ± 2.0	*p = 0.0003* *
P50 latency (ms)	49.4 ± 4.8	54.3 ± 8.9	*p = 0.0022* *
N95 latency (ms)	101.5 ± 11.4	93.8 ± 15.8	*p = 0.0244* *

* Statistically significant results are marked with an asterisk.

**Table 4:** A comparison of optical coherence tomography (OCT) parameters between GO patients with no clinical evidence of DON (Groups 1 and 2) and patients with confirmed DON (Group 3).

Parameter	GROUPS 1 and 2 NON-DON (Mean ± SD)	GROUP 3 DON (Mean ± SD)	*p-Value*
Average RNFL (µm)	108.2 ± 9.6	99.3 ± 17.2	*p = 0.0069* *
Superior RNFL (µm)	107.0 ± 10.2	98.2 ± 21.4	*p = 0.0178* *
Inferior RNFL (µm)	109.4 ± 11.8	100.4 ± 14.7	*p = 0.0137* *
Average GCC (µm)	95.7 ± 5.9	83.8 ± 7.9	*p < 0.0001* *
Superior GCC (µm)	95.1 ± 6.5	84.1 ± 9.4	*p < 0.0001* *
Inferior GCC (µm)	96.5 ± 6.0	83.6 ± 8.7	*p < 0.0001* *
FLV (%)	0.7 ± 0.9	3.6 ± 3.8	*p < 0.0001* *
GLV (%)	3.7 ± 3.2	14.6 ± 7.4	*p < 0.0001* *

* Statistically significant results are marked with an asterisk.

**Table 5:** A comparison of VEP components between GO patients with moderate-to-severe GO (Group 2) and patients with mild GO (Group 1).

Parameter	Check Size	Group 2 (Mean ± SD)	Group 1 (Mean ± SD)	*p-Value*
P100 latency (ms)	*0.4°*	96.6 ± 9.0	94.7 ± 8.2	*p = 0.3427*
	*0.9°*	96.2 ± 9.4	92.1 ± 6.6	*p = 0.0443* *
	*1.5°*	95.5 ± 12.0	92.0 ± 8.8	*p = 0.1770*
	*2.5°*	94.9 ± 14.6	93.0 ± 9.5	*p = 0.5562*
P100 amplitude (µV)	*0.4°*	9.4 ± 3.9	14.5 ± 7.0	*p = 0.0001* *
	*0.9°*	8.6 ± 3.6	13.6 ± 7.0	*p = 0.0002* *
	*1.5°*	7.9 ± 3.5	12.2 ± 5.5	*p = 0.0002* *
	*2.5°*	7.6 ± 3.6	10.8 ± 3.9	*p = 0.0014* *

* Statistically significant results are marked with an asterisk.

**Table 6:** A comparison of OCT parameters between GO patients with moderate-to-severe GO (Group 2) and patients with mild GO (Group 1).

Parameter	Group 2 (Mean ± SD)	Group 1 (Mean ± SD)	*p-Value*
Average GCC (µm)	94.5 ± 5.6	97.6 ± 5.9	*p* = 0.0282
Superior GCC (µm)	93.6 ± 6.2	97.4 ± 6.3	*p* = 0.0138

## Data Availability

Not applicable.
